# Effect of Interface Pretreatment of Al Alloy on Bonding Strength of the Laser Joined Al/CFRTP Butt Joint

**DOI:** 10.3390/mi12020179

**Published:** 2021-02-11

**Authors:** Yiyun Ye, Qi Zou, Yinan Xiao, Junke Jiao, Beining Du, Yuezhan Liu, Liyuan Sheng

**Affiliations:** 1 School of Mechanical Engineering, Yangzhou University, Yangzhou 225127, China; yeyiyun@nimte.ac.cn; 2 Laboratory of Aero Engine Extreme Manufacturing Technology of Zhejiang Province, Ningbo 315201, China; zouqi@nimte.ac.cn (Q.Z.); casting2002@163.com (Y.L.); 3 PKU-HKUST ShenZhen-HongKong Institution, Shenzhen 518057, China; ynxiao_pkusz@yeah.net (Y.X.); bndu10s@alum.imr.ac.cn (B.D.)

**Keywords:** laser joining, CFRTP, micro-texturing, dissimilar joint, anodizing, bonding strength

## Abstract

In the present research, the carbon fiber reinforced thermoplastic (CFRTP) was laser joined with the Al alloy whose joining interface was pretreated by laser micro-texturing, anodizing, and hybrid of laser micro-texturing and anodizing. The surface morphology of the pretreated Al joining interface and bonding strength of the corresponding Al/CFRTP butt joint were investigated. The results show that the laser micro-texturing has fabricated the micro-pit or micro-furrow in the Al joining interface. With the increasing of laser scanning times, the size of the micro-pit or micro-furrow decreases, when the laser scanning distance is constant. The bonding strength of the Al/CFRTP butt joint with Al joining interface pretreated by micro-texturing fluctuates with the increasing of laser scanning distance and times, reaching the maximum value of 20 MPa at laser scanning distance of 0.1 mm and 1 time. The anodizing pretreatment has formed the Al_2_O_3_ oxide layer on the Al joining interface. The Al/CFRTP butt joint with Al joining interface pretreated by anodizing obtains the maximum bonding strength of 11 MPa at anodizing time of 10 min. The hybrid pretreatment of micro-texturing and subsequent anodizing fabricates the regular grid structure with smooth micro-furrow and micro-pit, while the hybrid pretreatment of anodizing and subsequent micro-texturing fabricates the Al joining interface with explosive micro-pit and micro-furrow. The bonding strength of the Al/CFRTP butt joint with hybrid-pretreated Al joining interface is relative better than that of the Al/CFRTP butt joint with anodizing-pretreated Al joining interface but almost lower than that of the Al/CFRTP butt joint with micro-texturing pretreated Al joining interface. Such results should be attributed to the surface morphology of the Al joining interface.

## 1. Introduction

With the increasing attention on environmental protection and energy conservation, lightweight design for automobiles becomes more attractive in the automotive industry [1]. Generally speaking, structural optimization, new material, and new manufacturing technology can be applied during the lightweight design, among which material replacement is thought as the most effective approach [2]. The carbon fiber reinforced thermoplastic (CFRTP) composite, with a tensile strength of up to 2000 MPa along the fiber direction, a density of about 1.55 g/cm^3^, and excellent fatigue and corrosion resistance, has been considered as one of the potential materials used as structural components in automotive, aviation, and drones [3,4,5]. However, the CFRTP is a kind of polymer-based composite and its well joining with the metal framework of the automobile is still a problem, which handicaps its wide applications [6]. Therefore, to fully exploit the potential of the CFRTP composite, it is necessary to investigate the technology of joining CFRTP composite and metal.

Among the metals used in automotive industry, the aluminum (Al) alloys are the most attractive one, because of its good formability, excellent corrosion resistance, relative high strength, and relative low density [7,8]. Moreover, the proportion of Al alloy applied in the automotive increases gradually [9,10]. Then, how to well joining the Al alloy and CFRTP would become an issue in the future automotive industry [11]. Presently, the common methods to join the metal and CFRTP includes mechanical joining by riveting, adhesive bonding, and welding (thermal joining) [12]. The mechanical joining could provide the high bonding strength, but it destroys the original carbon fiber structure and results in the stress concentration, which is detrimental to the fatigue properties of the joint [13]. Moreover, the additional fasteners, including screws, bolts, and rivets, would increase the weight of the components [14,15]. The adhesive joining is convenient to operate and low-cost, but its environmental reliability is not good [16]. The performance of the adhesive joint drops sharply in the humid and hot environment, while the curing time of the adhesive is long [17,18]. To conquer these shortcomings, new methods of ultrasonic welding [19], resistance heating welding [20], friction stir welding [21], and laser welding [22] have developed and studied.

Laser welding is the most promising method, because of the advantages of non-contacting, easy automation, high flexibility, and high efficiency, and lots of researches have been performed in recent years [23,24,25]. Jung used laser heat conduction welding method to join carbon fiber reinforced polymer (CFRP) and metals such as the stainless steel, Al alloy, and galvanized steel [26,27]. They demonstrated that the variation of joining parameters exerted obvious influence on the joint quality. Roesner used a pulsed laser to process the surface of the metal material into a groove with a width of 40 μm and a depth of 50 μm [28]. Additionally, the shear strength of the joint between the glass fiber reinforced thermoplastic composite material and the metal reached 24 MPa. Lambiase realized the connection between metal and peek and the strength of the joint reached 30 MPa, which reached 53% of the poly-ether-ether-ketone (PEEK) shear strength [29]. Zhang used different pretreatment methods on the metal surface to improve the strength of the metal-composite joints, including surface laser-texture treatment and anodizing treatment [30,31]. The strength of the joint with anodizing treatment was about eight times higher than that of the untreated joint. It also revealed that the porous structure by the anodizing improved the wettability of PA6 resin on A6061 alloy surface and increased the mechanical anchoring effect of CFRP. Jiao used laser pretreatment on the metal surface and added polyamide 6 (PA6) resin in the joint interface, which effectively improved the strength of the joint [32,33]. He also established a finite element simulation model to predict the temperature distribution during laser connection and proposed a laser stir welding method to reduce thermal damage during welding [34].

As mentioned above, the previous related investigations mainly focused on the laser joining metal and CFRP with overlapping structure and the surface pretreatment of metal. Actually, the research on the laser joining CFRTP and Al alloy with butt joint is really few. Its joining effect and influence factor still needed full investigation. Therefore, in the present research, the joining interface of 7075 Al alloy was pretreated by laser micro-texturing, anodizing, or the combination of laser micro-texturing and anodizing. Then, the Al alloys with different joining interface pretreatments were laser joined with the CFRTP to obtain butt joint. The pretreated surface and bonding strength of the Al/CFRTP butt joint were investigate to explore the better processing parameters.

## 2. Materials and Methods

### 2.1. Materials and Preparation

The materials used in the experiment were 7075 Al alloy and CFRTP. The 7075 Al alloy was mainly composed of three elements: Zn (5.1–6.1%), Mg (2.1–2.9%), and Al in balance (the alloy proportion above was weight percentage). The CFRTP material was composed of PA and carbon fiber, and the PA content was about 50%. The Al alloy and CFRTP sheet were cut into the size of 50 mm (length) × 25 mm (width) × 2 mm (height). The additive material to promote the formation of butt joint was PA film with the thickness of 40 μm. The joining surface of the CFRTP was polished by sandpaper and finally, ultrasonically cleaned in deionized water to remove debris particles. The surface of Al alloy was cleaned by ethanol to remove the oil stain before the surface pretreatment.

### 2.2. Material Surface Pretreatment

#### 2.2.1. Laser Micro-Texturing Pretreatment

**Figure 1:**
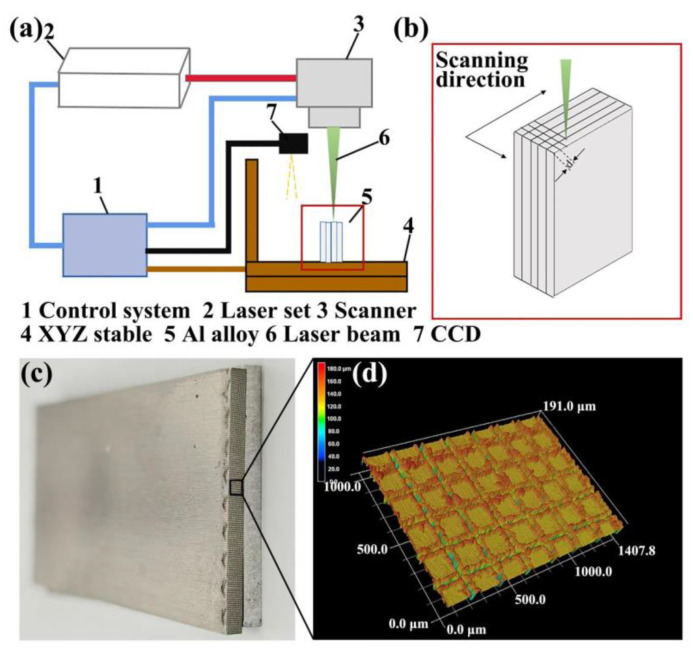
(**a**) Schematic of the picosecond laser processing system for laser micro-texture pretreatment, (**b**) the processing route of laser micro-texturing pretreatment, (**c**) the Al alloys with laser micro-texturing pretreated joining interface, and (**d**) typical morphology of the laser micro-texturing pretreated joining interface by laser scanning confocal microscope (LSCM) with magnification of 400×.

The picosecond laser processing (PLP) system was used to perform laser micro-texturing pretreatment on the Al alloy joining interface. The schematic of the PLP system was shown in Figure 1a. The laser scanned the Al alloy joining interface along the designed routes to form grid structures, as shown in Figure 1b–d. The model of the laser is edgewavewx4000lll-a, and the laser controller model is Aerotech a3200 (AEROTECH, Pittsburgh, PA, USA). The control platform is Aerotech ANT180, and the scanning galvanometer model is Aerotech AGV-14HP. The wavelength, pulse width, frequency, and power of the laser were 532 nm, 10 ps, 20 kHz, and 12 W, respectively. During the micro-texture pretreatment, the laser scanned the joining interface of the Al alloy with set routes to obtain the micro-texture. To investigate the effect of micro-texture on bonding strength of the Al/CFRTP butt joint, the laser scanning distance d (the distance between the laser-induced microgrooves) and the laser scanning time n were changed. The 9 specimen groups with different joining interface structure were obtained and labeled, as shown in Table 1. The MS represents the micro-texturing with small laser scanning distance and the MM represents the micro-texturing with medium laser scanning distance, while the MB represents the micro-texturing with big laser scanning distance. The following number represents the laser scanning times. The surface roughness of the pretreated Al interface was measured by the KEYENCE vk-x200k LSCM (KEYENCE, Osaka, Japan). Five positions at 400× magnification were analyzed to obtain the average value.

#### 2.2.2. Anodizing Pretreatment

**Figure 2:**
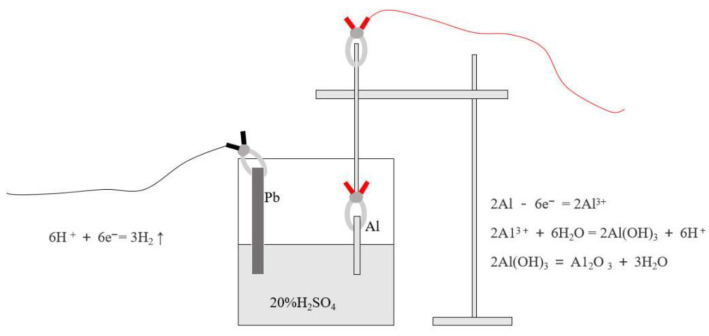
Schematic of the anodizing pretreatment on joining interface of the Al alloy.

The electrochemical workstation was used to anodize the joining interface of Al alloy. The schematic and reactions were shown in Figure 2. The lead was used as cathode in the electrochemical workstation and the Al alloy was set as anode. The H_2_SO_4_ solution with concentration of 20% and volume of 500 mL was used as electrolyte for each anodization. The stable DC voltage is 20 V and the maximum current does not exceed 2 A. The specimens with anodizing time of 5, 10, 15, and 20 min were labeled as A5, A10, A15, and A20, respectively.

#### 2.2.3. Hybrid Pretreatment of Laser Micro-Texturing and Anodizing

After the laser micro-texturing and anodizing pretreatments and following laser joining analysis, the best processing parameters of laser micro-texturing pretreatment and anodizing pretreatment were determined. Then the laser micro-texturing and anodizing were combined by application of their best processing parameters. In one experiment, the Al alloy was processed firstly by laser micro-texturing and then by anodizing, which was labeled as C1. In the other experiment, the Al alloy was processed firstly by anodizing and then by laser micro-texturing, which was labeled as C2.

### 2.3. Laser Joining

**Figure 3:**
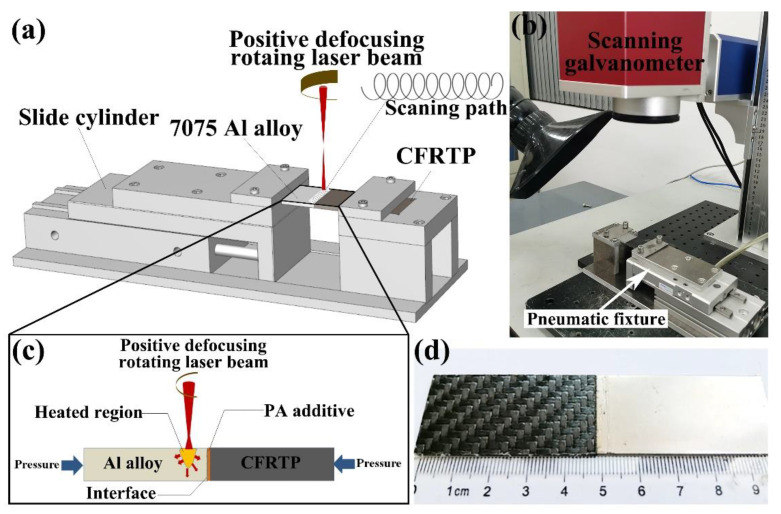
(**a**) Schematic of the laser joining Al/carbon fiber reinforced thermoplastic (CFRTP) butt joint, (**b**) the laser joining equipment and specimen fixture, (**c**) the schematic Al/CFRTP butt joint and its laser joining, and (**d**) the image of laser joined Al/CFRTP butt joint.

The schematic of laser joining Al/CFRTP butt joint is shown in Figure 3a. A low-power pulsed infrared laser was applied to join the Al alloy and CFRTP. The laser welding equipment includes the laser machine (Raycus RFL-P100M, RAYCUS, Wuhan, China), a control platform (Advantech 610L), and a scanning galvanometer (Sunny Technology 9210D, SUNNY, Beijing, China). With this scanning galvanometer, the laser beam can scan in a circle path. In addition, the rotational welding can be achieved. The power, wavelength, pulse width and pulse frequency of the low-power pulsed infrared laser were 100 W, 1064 nm, 72 ns, and 20 kHz, respectively. In order to reduce laser damage to Al alloy, the laser beam is defocused by 5 mm. The laser scanning speed, rotation radius, and rotation distance were set as 6 mm/s, 0.5 mm, and 0.05 mm, respectively. Before the laser joining, the Al alloy sheet with joining interface pretreatment and CFRTP sheet were clamped in the specimen fixture with impact pressure of 0.1 MPa, as shown in Figure 3b. During the laser joining, the laser beam scanned the Al alloy region adjacent to joining interface, which generated heat and conducted into the joining interface to melt the PA additive and the PA in CFRTP, as shown in Figure 3c. The molten PA flows into the micro-texture of the Al alloy joining interface under the pressure of the specimen fixture. Thereafter, the joined Al/CFRTP butt joint was air cooled to the room temperature. The as-fabricated Al/CFRTP butt joint was shown in Figure 3d. The Al/CFRTP butt joint fabricated from the untreated Al alloy is labeled as U.

### 2.4. Surface Characterization and Bonding Strength

The morphology of the Al alloy joining interface with laser micro-texture or anodizing pretreatment was observed by the KEYENCE VX-X200 (KEYENCE, Osaka, Japan) laser scanning confocal microscope (LSCM) and Phenom Pro scanning electron microscope (SEM) with the energy-disperse spectroscopy (EDS). The tensile test was performed on the CMT5105 electronic universal testing machine to obtain the bonding strength of the Al/CFRTP butt joint. The size of the tensile specimen is 100 mm (length) × 25 mm (width) × 2 mm (height), which is clamped by manual clamps with clamping areas of 25 mm (length) × 25 mm (width). The tensile test was carried out in air with a constant tensile speed of 2 mm/min at room temperature. Three specimens of same condition were tested to obtain the bonding strength. The Al/CFRTP butt joint and its fracture surface after tensile test were observed by SEM.

## 3. Results and Discussion

### 3.1. The Al/CFRTP Butt Joint with Laser Micro-Texturing Pretreatment

**Figure 4:**
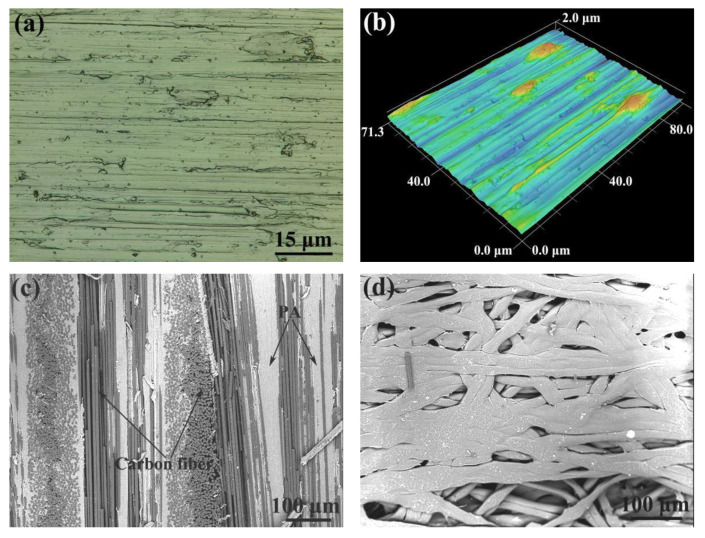
(**a**) Optical microscopy image of the untreated Al alloy, (**b**) surface morphology of the untreated Al alloy, (**c**) microstructure of the CFRTP, and (**d**) microstructure of the polyamide (PA) additive.

The surface morphology of the untreated Al alloy is shown in in Figure 4a,b. Clearly, the Al alloy polished by 600# abrasive paper has relative flat surface with small furrows. The depth of the furrow differs a litter, and its scope is in 2 μm. The typical microstructure of PA-based CFRTP is shown in Figure 4c. The PA-based CFRTP is mainly composed of black-gray carbon fiber and white-gray PA matrix. The carbon fibers are overlapped layer by layer and most carbon fibers are packed and bonded together by the PA. The layer of carbon fiber is weaved as decussate structure and the thickness of the layer of carbon fiber is about 300 μm. Moreover, the distribution of PA in the CFRTP is not uniform and the interface of the carbon fiber layer prefers to be the vacancy of PA. The SEM analysis on the PA additive reveals that the wires of PA are overlapped randomly, and there is relative high porosity, as shown in Figure 4d. Based on the SEM image, the PA wire has the size of 20–30 μm in diameter.

**Figure 5:**
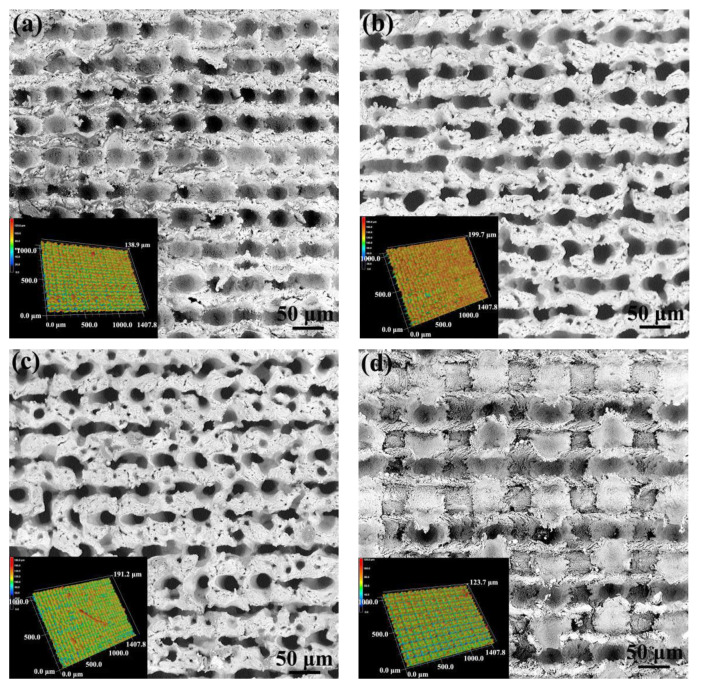

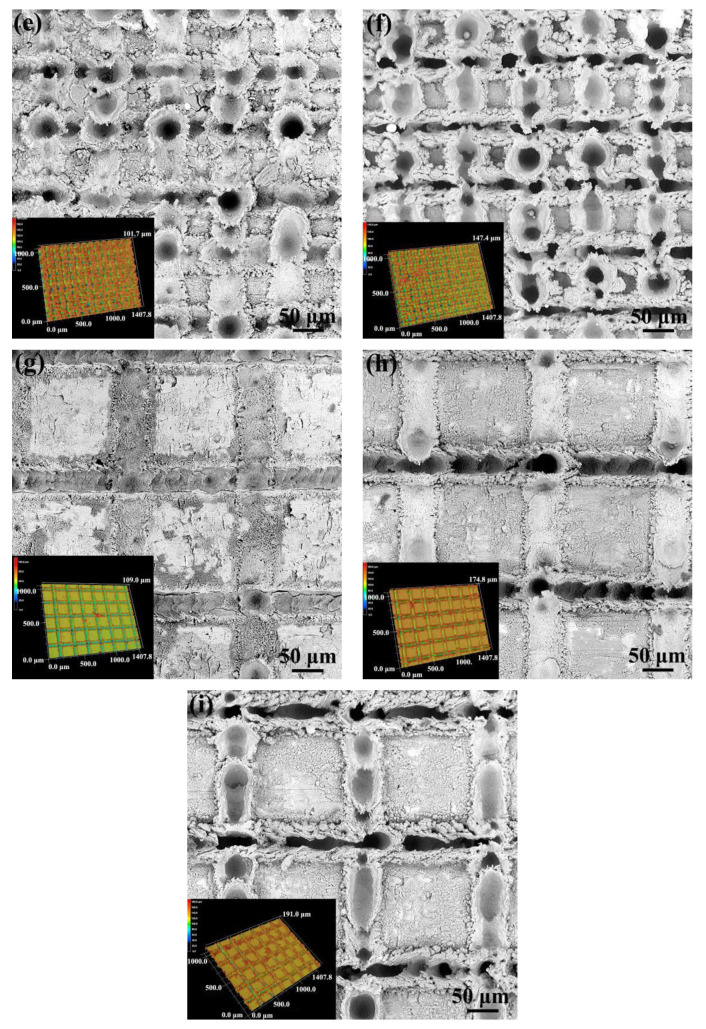
SEM micrograph and surface morphology of the Al alloy joining interface with different laser scanning distances and times: (**a**) d = 0.05 mm and n = 1, (**b**) d = 0.05 mm and n = 2, (**c**) d = 0.05 mm and n = 4, (**d**) d = 0.1 mm and n = 1, (**e**) d = 0.1 mm and n = 2, (**f**) d = 0.1 mm and n = 4, (**g**) d = 0.2 mm and n = 1, (**h**) d = 0.2 mm and n = 2, and (**i**) d = 0.2 mm and n = 4 (inset image showing the 3D morphology analyzed by LSCM with magnification of 400×).

The morphology of the Al interface processed by laser micro-texturing is shown in Figure 5. It can be found that the laser micro-texturing has resulted in regularly arranged pits in the Al joining interface. Moreover, the size and density of the pits are influenced by the laser scanning parameters obviously. When the laser scanning distance is about 0.05 mm, the depth of the pit increases obviously with the increasing of laser scanning times from 1 to 2, as shown in Figure 5a,b. However, the depth of pit decreases a little but the diameter of the pit decreases obviously when the laser scanning times increases from 2 to 4, as shown in Figure 1b,c. In addition, the former laser linearly scanned region would be affected by the later laser scanning with perpendicular direction. When the laser scanning distance is 0.1 mm, the evolution of pits in depth and diameter with the increasing of laser scanning times almost exhibits the similar tendency as that of the specimen with the laser scanning distance of 0.05 mm, as shown in Figure 5e,f. However, the average diameter of the pits in the specimens with laser scanning distance of 0.1 mm is a little bigger than that in the specimens with the laser scanning distance of 0.05 mm, when the laser scanning times is the same. When the laser scanning distance increases to 0.2 mm, the morphology of the Al joining interface changes from the densely arranged pit to the grid structure with pit or furrow distributed in the fringe, as shown in Figure 5g–i. The depth of the pit or furrow and the diameter of the pit increases with the increase in laser scanning times. Such surface morphology evolution should be attributed to the interaction between the perpendicular routes during laser scanning. When the laser scanning distance is small, the later laser scanning would overlap the former laser scanning routes partly, which weakens the micro-texturing. When the scanning distance is high, the weakening from the interaction would decrease, because the overlap of perpendicular laser scanning routes decreases obviously.

**Figure 6:**
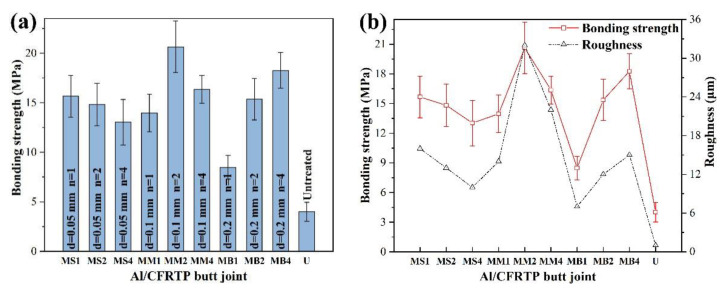
(**a**) Bonding strength of the Al/CFRTP butt joint with different laser micro-texturing Al joining interface and (**b**) variation of surface roughness and bonding strength of Al alloy pretreated by different laser micro-textures.

The bonding strength of the Al/CFRTP butt joint with different Al interface pretreatment is shown in Figure 6a. Obviously, the bonding strength of the Al/CFRTP butt joint with laser micro-texturing pretreatment is higher than that of the Al/CFRTP butt joint with untreated Al alloy. However, for the Al/CFRTP butt joints with laser micro-texturing pretreatments, the bonding strength still fluctuates with the pretreatment parameter. When the laser scanning distance is 0.05 mm, the bonding strength of the Al/CFRTP butt joint decreases gradually with the increasing of laser scanning times. When the laser scanning distance increases to 0.1 mm, the bonding strength of the Al/CFRTP butt joint increases again with the increasing laser scanning times and reaches the maximum value at the 2 times laser scanning. Thereafter, the bonding strength begins to drop. When the laser scanning distance increases to 0.2 mm, the bonding strength of the Al/CFRTP butt joint reaches its minimum value at the 1 time laser scanning. With the further increase in laser scanning times, the bonding strength of the Al/CFRTP butt joint increases sharply. To study the relationship between the surface morphology and bonding strength, the bonding strength of the Al/CFRTP butt joint and roughness of the Al joining interface were comparatively analyzed, as shown in Figure 6b. LSCM was used to measure the roughness for five positions at 400× magnification to obtain the average value. Generally, the roughness of the Al joining interface decreases with the laser scanning times when the laser scanning distance is 0.05 mm. When the laser scanning distance is 0.1 mm, the roughness of the Al joining interface increases firstly and then drops. When the laser scanning distance is 0.2 mm, the roughness of the Al joining interface increase gradually. It can be found that the binding strength almost changes with the roughness synchronously. Then, it can be deduced that the bonding strength of the Al/CFRTP butt joint is related with the roughness of the Al joining interface directly.

**Figure 7:**
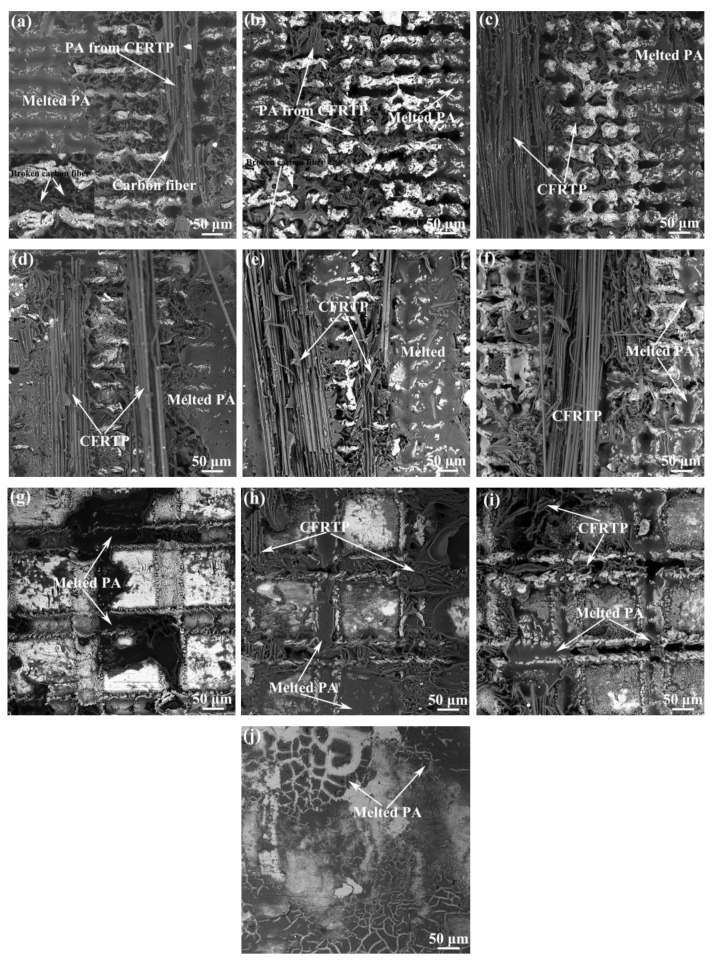
Fracture surface of the Al/CFRTP butt joint with different pretreated Al joining interface: (**a**) d = 0.05 mm and n = 1 (inset image showing the broken carbon fiber), (**b**) d = 0.05 mm and n = 2 (inset image showing the broken carbon fiber), (**c**) d = 0.05 mm and n = 4, (**d**) d = 0.1 mm and n = 1, (**e**) d = 0.1 mm and n = 2, (**f**) d = 0.1 mm and n = 4, (**g**) d = 0.2 mm and n = 1, (**h**) d = 0.2 mm and n = 2, (**i**) d = 0.2 mm and n = 4, and (**j**) untreated (d: laser scanning distance, n: laser scanning times).

To explore the influence of the Al joining interface surface morphology on the bonding strength of the Al/CFRTP butt joint, the fracture surface of the Al/CFRTP butt joint after tensile test were characterized, as shown in Figure 7. Because the delamination in CFRTP is the main failure mode, the characterization is mainly performed on the Al alloy interface. Clearly, the delaminated CFRTP and fractured PA are the main characteristics in the fracture surface, but the ratio and amount are different. When the laser scanning distance in Al joining interface is 0.05 mm, the morphology of the fractured PA and ratio of the ration of delaminated CFRTP are almost similar, as shown in Figure 7a–c. The fractured carbon fiber with PA could be observed in the pits, which indicates that such a laser micro-texturing structure could promote the mechanical interlocking between the Al alloy and CFRTP. When the laser scanning distance in Al joining interface is 0.1 mm, the fracture surface of the Al/CFRTP butt joint exhibits more laminated CFRTP, as shown in Figure 7d–f. That indicates that the CFRTP has better bonding with the Al alloy. Moreover, the pits filled with melted PA could be observed on the Al joining interface, which indicates that such a surface morphology is beneficial to the spreading of the melted PA. When the laser scanning distance in Al joining interface is 0.2 mm, the fracture surface exhibit less melted PA and delaminated CFRTP, as shown in Figure 7g–i. Especially, in the Al joining interface with 1 time laser scanning, only small region has covered the melted PA. With the increasing laser scanning times, more melted PA mainly distributed in the furrow or pit could be observed. These phenomena indicate that the Al joining interface with reasonable size of pit or furrow would be more important for the bonding strength of the Al/CFRTP butt joint. The fracture surface of the Al/CFRTP butt joint with untreated Al alloy is also exhibited in the Figure 7j. It could be found that the melted PA is spread on the Al joining interface inhomogeneously with some cracks inside. Then it is understandable that the Al/CFRTP butt joint with untreated alloy has the lowest bonding strength.

### 3.2. The Al/CFRTP Butt Joint with Anodizing Pretreatment

**Figure 8:**
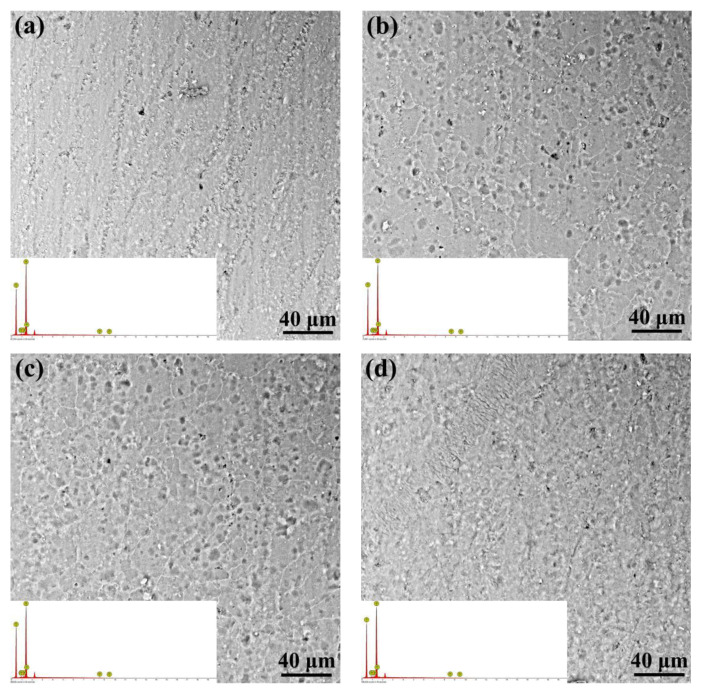
Surface morphology of the Al alloy joining interface with different anodizing pretreatment: (**a**) anodizing time of 5 min, (**b**) anodizing time of 10 min, (**c**) anodizing time of 15 min, and (**d**) anodizing time of 20 min (inset image showing the energy-disperse spectroscopy (EDS) of the corresponding surface).

The surface morphology of the Al joining interface with anodizing pretreatment is shown in Figure 8. Clearly, the anodizing pretreatment has resulted in a smoother surface for the Al joining interface. The EDS analyses on the surfaces of all anodizing pretreated Al alloy reveal that all surfaces have been covered by the Al_2_O_3_ oxide layer. According to the previous research [35], the thickness of the Al_2_O_3_ oxide layer would increase with the increase in the anodizing time. In the present research, it can be found that the microstructure of the anodizing pretreated surface has experienced obvious evolution. When the anodizing time changes from 5 to 10 min, the grain size of Al_2_O_3_ increases from several micrometers to dozens of micrometers, as shown in Figure 8a,b. When the anodizing time increases to 15 min, the grain size of Al_2_O_3_ almost have no change. It seems that the grain size of Al_2_O_3_ decreases a little, when the anodizing time is 20 min. Such microstructure evolution should be attributed to the anodizing time, which determines the crystal growth of Al_2_O_3_.

**Figure 9:**
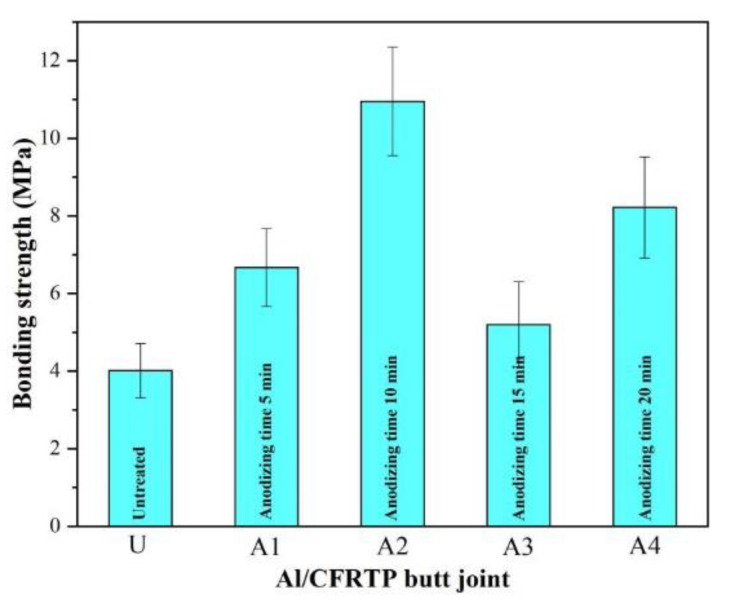
Variation of the bonding strength of the Al/CFRTP butt join with different anodizing pretreated Al interface.

The bonding strength of the Al/CFRTP butt joint with different anodizing pretreated Al joining interface is shown in Figure 9. Compared with the Al/CFRTP butt joint with untreated Al alloy, the anodizing pretreatment has improved the bonding strength of the Al/CFRTP butt joint. However, the anodizing time still exert obvious influence on the bonding strength. With the increase in anodizing time, the bonding strength of the Al/CFRTP butt joint increases firstly and reaches the maximum value at time of 10 min. Then, the bonding strength of the Al/CFRTP butt joint decreases and reaches the minimum value. Thereafter, the bonding strength of the Al/CFRTP butt joint increases again.

**Figure 10:**
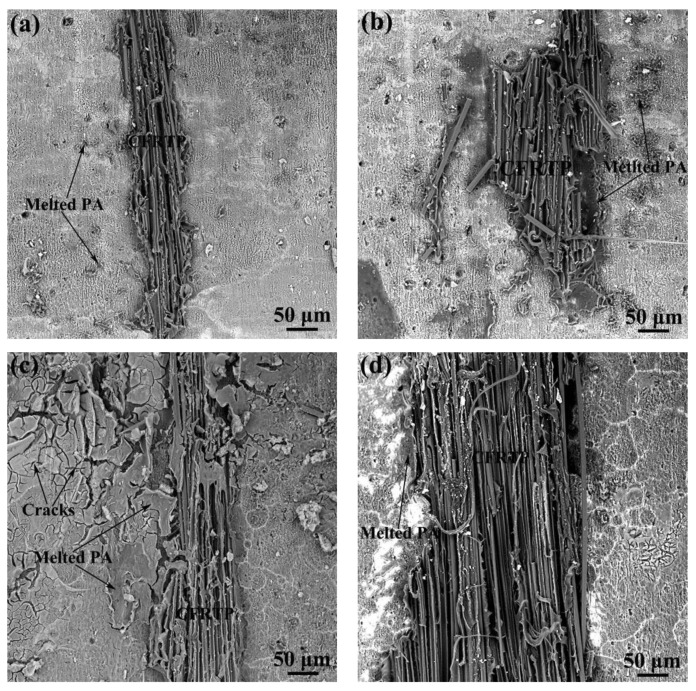
Surface morphology of the different anodizing pretreated butt joint interface after the tensile test: (**a**) anodizing time of 5 min, (**b**) anodizing time of 10 min, (**c**) anodizing time of 15 min, and (**d**) anodizing time of 20 min.

To explore the effect of anodizing pretreatment on bonding strength, the fracture surface of the Al/CFRTP butt joint with different anodizing pretreatment is observed, as shown in Figure 10. Clearly, small residual CFRTP on Al joining interface is the main characteristic, but the amount of the residual CFRTP is different. For the Al/CFRTP butt joints with the Al joining interface anodized for 5 and 15 min, their amount of residual CFRTP left on fracture surface is almost the same and less than that on the Al joining interface with anodizing pretreatment of 10 and 20 min, as shown in Figure 10a,c. However, the cracks in the Al_2_O_3_ oxide layer could be found in the Al joining interface with anodizing of 15 min. For the Al/CFRTP butt joints with the Al joining interface anodized for 15 and 20 min, more bonded CFRTP and melted PA could be observed on the Al joining interface, as shown in Figure 10b,d. In addition, the microcracks could be observed on the Al joining interface with 20 min anodizing pretreatment. Then, it could be understood that the bonding strength of the Al/CFRTP butt joint with the Al joining interface anodized for 15 min decreases, because the formation of crack in the Al_2_O_3_ oxide layer promotes the failure of the butt joint. According to research [36], the reasonable surface morphology also could increase the bonding strength, so the Al/CFRTP butt joint with the relative coarse Al joining interface and no crack has the highest bonding strength.

### 3.3. The Al/CFRTP Butt Joint with Micro-Texturing and Anodizing Hybrid Pretreatment

**Figure 11:**
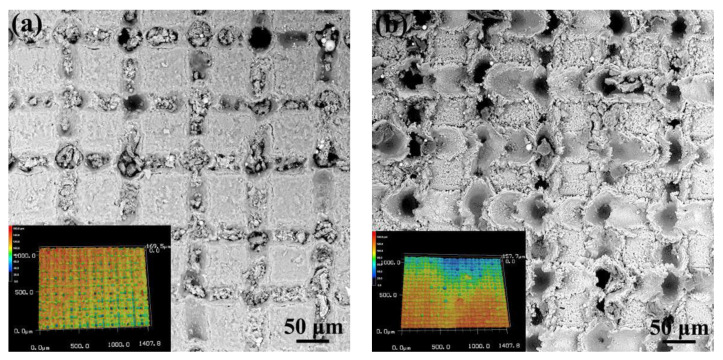
Surface morphology of the Al alloy joining interface pretreated by anodizing of 10 min and subsequent laser micro-texturing (**a**) and laser micro-texturing and subsequent anodizing of 10 min (**b**) (inset image showing the 3D morphology analyzed by LSCM with magnification of 400×).

The surface morphology of the Al joining interface with hybrid pretreatment of micro-texturing and anodizing is shown in Figure 11. It can be found the hybrid pretreatment with different order has resulted in obviously different surface morphology. The Al joining interface pretreated by micro-texturing and subsequent anodizing has distinct grid structure with furrow along fringes, as shown in Figure 11a. The 3D morphology analysis reveals that the depth of the furrow is about 170 μm. Moreover, the small oxide particles have filled in the furrows. When the Al joining interface is pretreated by anodizing and subsequent micro-texturing, the surface exhibit the muddy-road like morphology, as shown in Figure 11b. The subsequent laser micro-texturing pretreatment has resulted in the explosive pits on the laser linearly scanned intersection points, which forms the fragments adjacent to the explosive pits. Moreover, the subsequent laser micro-texturing pretreatment has destroyed the continuity of the Al_2_O_3_ oxide layer formed during the anodizing pretreatment.

**Figure 12:**
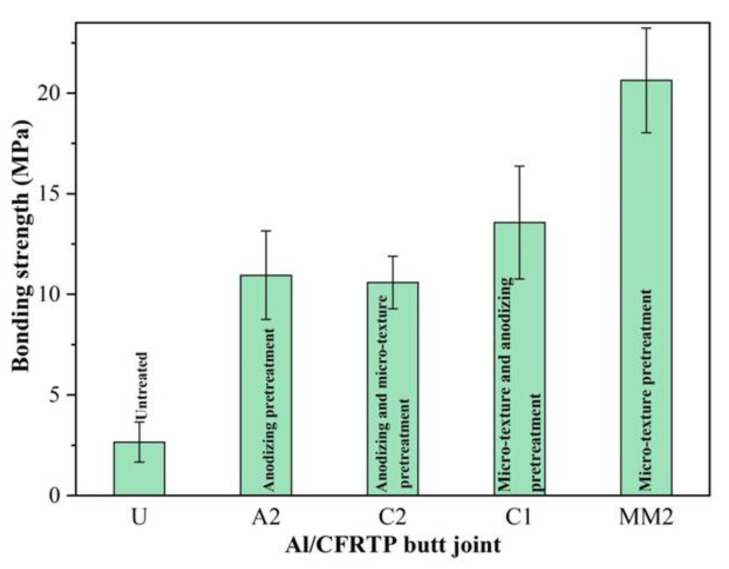
Bonding strength of the Al/CFRTP butt joints with different Al joining interface pretreatments.

The bonding strength of the Al/CFRTP butt joints with Al joining interface pretreated by hybrid pretreatments, untreated, typical anodizing, and micro-texturing are summarized in the Figure 12. It can be found that the bonding strength of the Al/CFRTP butt joint with Al joining interface pretreated by micro-texturing and subsequent anodizing is higher than that of the Al/CFRTP butt joint with Al joining interface pretreated by anodizing and subsequent micro-texturing. The Al/CFRTP butt joint with Al joining interface pretreated by anodizing and subsequent micro-texturing has almost the similar bonding strength as the Al/CFRTP butt joints with Al joining interface pretreated by anodizing for 10 min. However, the Al/CFRTP butt joints with Al joining interface pretreated by hybrid pretreatments have lower bonding strength, compared with the Al/CFRTP butt joint with Al joining interface pretreated by micro-texturing (0.1 mm laser scanning distance and 1 time laser scanning).

**Figure 13:**
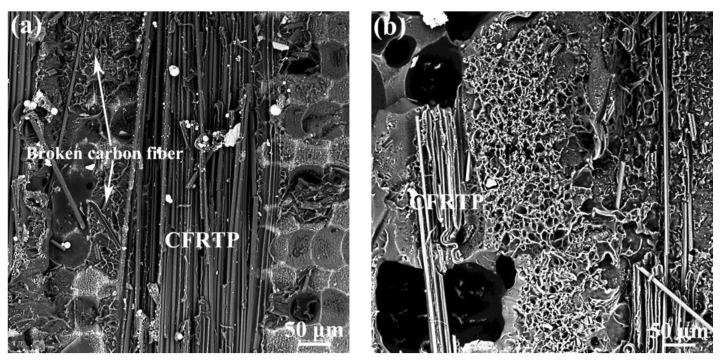
Fracture surface of the Al/CFRTP butt joints with Al joining interface pretreated by different hybrid pretreatments: (**a**) micro-texturing and subsequent anodizing and (**b**) Anodizing and subsequent micro-texturing.

The fracture surface of the Al/CFRTP butt joint with Al joining interface pretreated by hybrid pretreatments are shown in Figure 13. On both specimens, the delaminated CFRTP and fractured PA could be observed but the fracture surface of the Al/CFRTP butt joint with Al joining interface pretreated by micro-texturing and subsequent anodizing has more delaminated CFRTP, as shown in Figure 13a,b. In addition, there are more broken carbon fibers in the residual PA bonded in the fracture surface of the Al/CFRTP butt joint with Al joining interface pretreated by micro-texturing and subsequent anodizing. However, there is still many residual PA in the fracture surface of the Al/CFRTP butt joint with Al joining interface pretreated by anodizing and subsequent micro-texturing, but a few broken fibers could be observed in the residual PA. Such a phenomenon indicates that the Al joining interface pretreated by micro-texturing firstly and anodizing lastly could bond more PA, which increases the adhesion of the carbon fibers.

**Figure 14:**
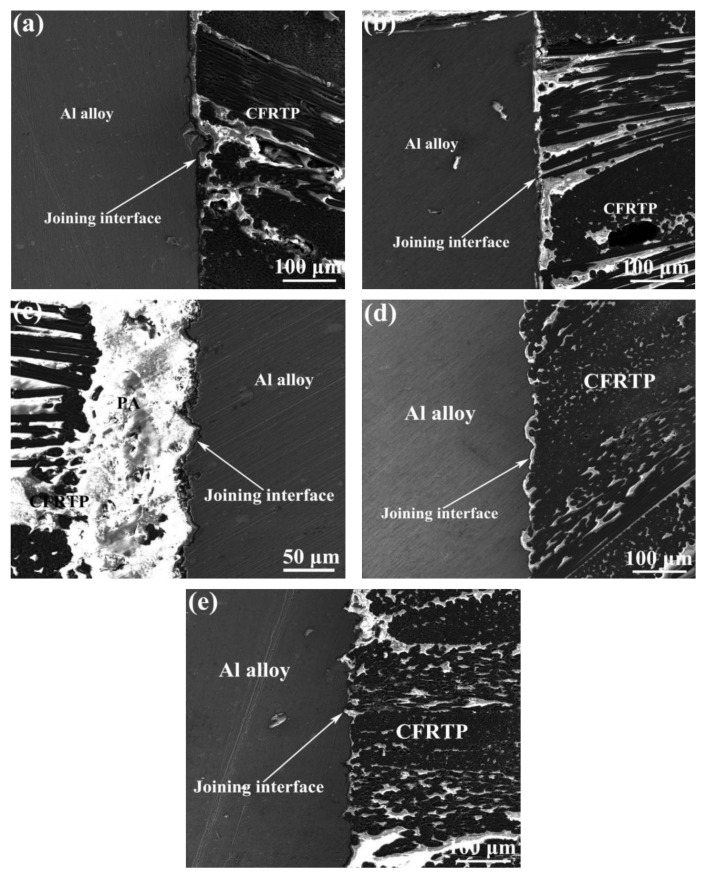
Microstructure of the Al/CFRTP butt joint interface with different pretreatments: (**a**) untreated, (**b**) anodizing of 10 min, (**c**) anodizing of 10 min subsequent followed with laser micro-texture, (**d**) laser micro-texture subsequent followed with anodizing of 10 min, and (**e**) laser micro-texture with scanning distance of 0.1 mm and 1 time laser scanning.

To explore the bonding mechanism, the typical Al/CFRTP butt joint interfaces with different pretreatments on Al joining interfaces were observed and the results are shown in Figure 14. The relative straight interface could be found in Al/CFRTP butt joints with untreated Al alloy and the Al joining interface pretreated with anodizing. The difference is that the anodizing pretreated Al joining interface has a thin film, which connects the Al alloy and the CFRTP. The Al/CFRTP butt joint with Al joining interface pretreated by anodizing and subsequent micro-texturing has the highest fluctuated Al joining interface and the thickest PA between CFRTP and Al alloy. For the Al/CFRTP butt joints with Al joining interface pretreated by micro-texturing and hybrid pretreatment of micro-texturing and subsequent anodizing, their interfaces exhibit the similar morphology with regular fluctuation. However, there is a continuous and thin Al_2_O_3_ oxide transition layer, and the sinking place is relatively smooth in the joint with hybrid pretreatment of micro-texturing and subsequent anodizing, which is beneficial to the adhesion of CFRTP. For the Al/CFRTP butt joint with Al joining interface pretreated by micro-texturing, its sinking place is sharp and there is no continuous transition layer.

According the recent research [33,37], the bonding strength of the laser-joined metal and carbon fiber reinforced polymer-based composite is obviously related with the interface morphology. The reasonable metal surface would benefit the mechanical interlocking between the metal and carbon fiber-reinforced polymer-based composite. In the present research, the laser micro-texturing processes the Al joining interface with micro-pit or micro-furrow, and the results also demonstrates that the reasonable distribution and size of the micro-pit or micro-furrow is beneficial to the spreading of the melted PA and its filling, which contributes to the bonding strength. Therefore, the Al/CFRTP butt joint with Al joining interface pretreated by laser scanning distance of 0.1 mm and 1 time laser scanning obtains the best bonding strength. However, the bonding strength of Al/CFRTP butt joint mainly origins from the mechanical interlocking between the Al alloy and CFRTP. Though the following anodizing pretreatment could promote the molecular bonding between the Al_2_O_3_ oxide layer and PA matrix of CFRTP, the effect is still weaker than the mechanical interlocking [38,39]. Therefore, the bonding strength of the best Al/CFRTP butt joint with Al joining interface pretreated by anodizing is still lower than that of the best Al/CFRTP butt joint with Al joining interface pretreated by micro-texturing. The hybrid pretreatment has combined the advantages of the anodizing and micro-texturing, but anodizing after micro-texturing has smoothed the micro-pit or micro-furrow, which decreases the mechanical interlocking between the CFRTP and Al alloy. Therefore, the bonding strength of the Al/CFRTP butt joint with Al joining interface pretreated by micro-texturing and subsequent anodizing just increases a little, compared with the Al/CFRTP butt joint with Al joining interface pretreated by anodizing. The micro-texturing after anodizing almost destroys the continuity of the oxide layer on the Al interface. Moreover, the highly concentrated heat scanning and shock would influence the interface structure between the oxide layer and Al alloy, which is detrimental to the interfacial strength [40,41]. Then, the bonding strength of the Al/CFRTP butt joint with Al joining interface pretreated by anodizing and subsequent micro-texturing is a little lower than that of the Al/CFRTP butt joint with Al joining interface pretreated by anodizing for 10 min. Considering the influence of the Al joining interface, the interface morphology and interfacial film structure still needs to be optimized in future work. The effect of morphology, dimension, and distribution of micro-texture on the bonding strength of the Al/CFRTP butt joint should be studied systematically. Moreover, the interfacial film with reasonable morphology and structure should be explored to improve the bonding strength of the Al/CFRTP butt joint. In addition, the optimal combination of the surface morphology processing and interfacial film should be studied further.

## 4. Conclusions

In the present research, the surface pretreatment had been carried out on the Al joining interface by laser micro-texturing, anodizing, and hybrid of laser micro-texturing and anodizing. The surface morphology of the pretreated Al joining interface and bonding strength of the corresponding Al/CFRTP butt joint were investigated and some conclusion could be drawn as following.

1. The laser micro-texturing has processed the micro-pit or micro-furrow in the Al joining interface. With the increasing of laser scanning times, the size of the micro-pit or micro-furrow decreases, when the laser scanning distance is defined. With the increasing of laser scanning distance and time, the bonding strength of the Al/CFRTP butt joint firstly decreases gradually and then increases. After reaching the maximum value of 20 MPa at laser scanning distance of 0.1 mm and 1 time laser scanning, the bonding strength of the Al/CFRTP butt joint decreases again and reaches the minimum value at laser scanning distance of 0.2 mm and 1 time laser scanning, and then, the bonding strength of the Al/CFRTP butt joint increases again.
2. The anodizing pretreatment has formed the Al_2_O_3_ oxide layer on the Al joining interface. With the increase in anodizing time, the bonding strength of the Al/CFRTP butt joint increases firstly and reaches the maximum value of 11 MPa at anodizing time of 10 min. The further increase in anodizing time would decrease the bonding strength of the Al/CFRTP butt joint and reach minimum value at anodizing time of 15 min. Thereafter, the bonding strength of the Al/CFRTP butt joint would increase again.
3. The hybrid pretreatment of micro-texturing firstly and anodizing lastly fabricates the regular grid structure with smooth micro-furrow and micro-pit, while the hybrid pretreatment of anodizing firstly and micro-texturing lastly processes the Al joining interface with explosive micro-pit and micro-furrow. The bonding strength of the Al/CFRTP butt joint with Al joining interface pretreated by hybrid pretreatment is relative better than that of the Al/CFRTP butt joint with Al joining interface pretreated by anodizing but almost lower than that of the Al/CFRTP butt joint with Al joining interface pretreated by micro-texturing. Such results should be attributed to the surface morphology of the Al joining interface.
4. Considering the influence of the Al joining interface, the interface morphology and interfacial film structure still needs to be optimized in future work. The effect of morphology, dimension, and distribution of micro-texture on the bonding strength of the Al/CFRTP butt joint should be studied systematically. Moreover, the interfacial film with reasonable morphology and structure should be explored to improve the bonding strength of the Al/CFRTP butt joint. In addition, the optimal combination of the surface morphology processing and interfacial film should be studied further.

## Figures and Tables

**Table 1:** Specimens with different laser micro-texturing pretreatment parameters.

Laser Scanning Distance d (mm)	Laser Scanning Times n		
	1	2	4
0.05	MS1	MS2	MS4
0.1	MM1	MM2	MM4
0.2	MB1	MB2	MB4

## Data Availability

Data available upon request from the corresponding author.
